# ^18^F- FDG PET/CT joint assessment of early therapeutic response in rheumatoid arthritis patients treated with rituximab

**DOI:** 10.1186/s41824-017-0022-y

**Published:** 2018-02-05

**Authors:** Pacôme FOSSE, Marie-Joelle KAISER, Gauthier NAMUR, Dominique de Seny, Michel G. MALAISE, Roland HUSTINX

**Affiliations:** 1 Division of Nuclear Medicine, University of Liège and CHU de Liège, Sart Tilman B35, 4000 Liège, Belgium; 2 Division of Rheumatology, University of Liège and CHU de Liège, Liège, Belgium; 3 Division of Nuclear Medicine, University Hospital of Angers, Angers, France

**Keywords:** ^18^F- FDG, PET/CT, Rheumatoid arthritis, Rituximab

## Abstract

**Background:**

^18^F–FDG PET/CT has been proposed in the evaluation of the disease activity in rheumatoid arthritis (RA). The goals of this study were to evaluate the reproducibility of the technique, to compare metabolic parameters to clinical, biological and ultrasonographic parameters before and after treatment and to evaluate whether the early metabolic response was related to the outcome. ^18^F- FDG PET/CT of the hands, wrists and knees was obtained in 15 patients with anti-TNFα refractory RA, at baseline and 16 weeks after treatment with rituximab. The number of PET-positive joints (PET+ joints), the cumulative standard uptake value (cSUV) and the composite index (CI) were defined. The composite clinical index DAS_28_, CRP serum levels and the number of joints positive at ultrasonography (US+ joints) and the cumulative synovial thickness (CST) were also assessed at baseline and week 24.

**Results:**

High interobserver agreement was observed, both at baseline and after treatment. The number of PET+ joints was not correlated with the number of joints tender or swollen. The 3 metabolic parameters were strongly correlated with US, CRP and DAS_28_ at baseline and with US and CRP (CSUV, CI) at week 16, but no longer with the DAS_28_ index. The metabolic response based on the change in the visual PET/CT joint analysis predicted the outcome with a high negative predictive value of 91%, with a 91% specificity, and an 86% accuracy.

**Conclusions:**

These preliminary data suggest that ^18^F- FDG PET/CT is a reproducible and accurate tool for evaluating disease activity in refractory rheumatoid arthritis and its non-response to rituximab. The correlation obtained with US joint assessment gives relevance to objective diseased joints through imaging techniques.

## Background

Rheumatoid arthritis (RA) is a frequent chronic systemic inflammatory disorder characterized by the development of a joint synovitis, which is responsible for pain and swelling. This finally leads to joint space narrowing and marginal erosions, reflect of cartilage and bone degradation. The morbidity is high, as among those affected, about 10% are no longer able to work one year after their condition is diagnosed, reaching as high as 50% after 10 to 20 years (Albers et al., 1999).

The therapeutic approach has been revolutionized by biotherapies, in particular the anti-TNF-α drugs that prevent bone destruction and cardiovascular morbidity, reduce the dependence on corticosteroids and improve quality of life (Weinblatt et al., 1999; Weinblatt et al., 2003). However, 20–30% of the anti-TNF-α-treated patients are considered as non-responders, at least clinically (O'Dell, 2004). In this context, new targeted therapies were developed based upon the complex pathogenesis of RA and the contribution made by B cells (Dorner & Burmester, 2003). Several clinical trials have demonstrated that rituximab is both effective and safe in anti-TNF-α-resistant RA patients with a response rate of 50–70% (Edwards et al., 2004; Cohen et al., 2006). However, treatment monitoring remains a challenge in rheumatological practice as the duration of the clinical response is not predictable and recent data suggest that systematic rituximab re-treatment at 6 months might be a beneficial approach compared to re-treatment upon disease flare (Vander Cruyssen et al., 2010).

Accordingly, identification of biomarkers or techniques predicting response to biologics in RA remains a relevant issue. Parameters such as the absence of Rheumatoid Factor or anti-CCP antibodies, multiple previous exposures to anti-TNF-α therapies, and high levels of circulating preplasma cell and subsequent incomplete depletion, have been recognized as predictors of treatment failure (Cohen et al., 2006; Thurlings et al., 2008; Vital et al., n.d.). However, these predictive parameters remain of limited interest in the daily practice and the decision whether to re-treat or not is far from evident.

We previously proposed ^18^F- FDG PET as a unique imaging technique that can assess the metabolic activity of synovitis and measure the disease activity in RA (Beckers et al., 2004). The number of PET-positive joints and the cumulative SUV were significantly correlated with the DAS_28_, which is a composite disease activity score that combines the swollen and tender joint counts, the erythrocyte sedimentation rate (DAS_28_-ESR) or C-reactive protein serum levels (DAS_28−_CRP) and the patient global assessment (Prevoo, 1995). The number of PET-positive joints and the cumulative SUV were also significantly correlated to the presence and the thickness of synovitis revealed by ultrasonography (Beckers et al., 2004; Beckers et al., 2006). Further, changes in the CRP and MMP-3 serum levels observed after a four-week period of treatment with an anti-TNF-α drug were significantly correlated with changes in SUV scores.

The purposes of this study were thus the following: (a) to evaluate the reproducibility of ^18^F- FDG PET/CT for assessing the activity of the disease; (b) to compare metabolic parameters to clinical, biological and ultrasonographic parameters before and after rituximab treatment; (c) to evaluate whether the metabolic response observed at week 16 could predict the outcome two months later (week 24).

## Methods

### Patients

This prospective study included 15 patients (12 women, 3 men) with active RA fulfilling the American College of Rheumatology 1987 revised criteria (Arnett et al., 1988). All patients were refractory to anti-TNFα treatments. The protocol was approved by our local Ethics Committee and an informed consent was obtained from all patients before participation in the study. The mean age was 53.8 years (range, 29–75) and the mean disease duration was 11.2 years (range, 2–38). Rituximab was administrated intravenously at dose of 1 g on days 1 and 15.

### Study design

^18^F- FDG PET/CT was performed 1 to 28 days before the first rituximab administration. All patients had a clinical, biological and ultrasonographic evaluation at baseline and 16 weeks after the first rituximab administration. The clinical status was again determined at week 24. The timing was dictated by regulatory requirements in Belgium at the time of the study initiation. Indeed the reimbursement of a second perfusion of rituximab was only granted when an objective response was recorded at the 16th week following the initial perfusion. The clinical status of each patient, the laboratory parameters C-reactive protein (CRP) and erythrocyte sedimentation rate (ESR) were measured on the same day. ESR was determined by the Westergren method and CRP levels measured by nephelometry. The DAS_28_-ESR (Prevoo, 1995) was established by the same study nurse in all patients and in all visits. US was performed by an experimented rheumatologist (M-JK) within one week of the clinical assessment and the ^18^F–FDG PET/CT analysis, and unaware of the results obtained.

### Ultrasonography

US was performed using a B-mode 13.0-MHz transducer using a procedure extensively described elsewhere (Beckers et al., 2004; Ribbens et al., 2003). Synovitis was defined as a thickness ≥ 1 mm. A joint was considered positive for US when synovial thickness reached that threshold. For each patient and at each time point, the cumulative synovial thickness was obtained by adding the individual joint measurements. The US did not show any indirect sign of osteoarthritis, such as osteophytes.

### ^18^F–FDG PET/CT

All image data were acquired using the Gemini Dual PET-CT scanner (Philips Medical systems). The GEMINI Dual is an open PET-CT system that combines a helical dual slice CT and a 3D PET scanner. Images were reconstructed using a 3D row action maximum likehood algorithm (RAMLA). Attenuation correction was applied using CT date (CTAC).

Patients fasted for approximately 6 h before the injection of an activity of 4 Mbq/kg. At baseline, the uptake time was 38 min (min) (range, 27–58) for wrists and hands and of 57.8 min (range, 42–78) for knees. At the 16th week, it was 41.6 min (29–75 min) for wrists and hands and 54.8 min (range, 49–74) for knees. There were no statistical differences between these values.

The images were first visually analyzed and joints were considered as positive for synovitis when the ^18^F–FDG uptake was increased compared to the background in areas corresponded to joint synovium on CT, i.e. either when thickened synovium was recognized on CT or in locations corresponding anatomically to synovium, excluding uptake in other structures such as muscle and tendons. The evaluation was performed by an independent experienced PET assessor (PF), who was unaware of the clinical and the sonographic status of the patients. PET assessments were performed on the knees, wrists, MCP and PIP for a total of 24 joints in each patient. The ^18^F–FDG uptake was then quantified using the maximum standardized uptake value (SUVmax). In PET-positive joints according to the visual analysis, the SUVmax was obtained by drawing a region of interest (ROI) over the most active synovial area identified. When no synovitis was identified, ROIs were placed in the corresponding areas on the CT: at the dorsal surface of the radius (on top of the lunate) for the wrists, over the lateral recess at the level of the midpatella for the knees and for the small joints (MCP, PIP), ROIs were drawn around the appropriate joint. A global metabolic assessment was obtained through the number of PET-positive joints (visual evaluation), the sum of all SUVs (cumulative SUV) and a composite index taking into account both parameters. The composite index (CI) is defined as follows: CI = cumulative SUV x (number of PET-positive joints/total number of joints evaluated). The first nine studies were independently read by two nuclear physicians (PF, GN) to evaluate the interobserver agreement.

### Statistical analysis

Interobserver agreement for identifying synovitis using visual analysis was assessed using the Cohen κ-test. For the semi-quantitative analysis (SUVmax), the concordance correlation coefficient was obtained. To compare PET data (visual and SUVmax) with the other parameters (tender joints, swollen joints, VAS, CRP, US), we used the Cohen κ-test or the Spearman’s rank-order correlation coefficient. *P* value ≤0.05 were considered significant.

## Results

The clinical, biological, ultrasonographic and metabolic data for all 15 patients are shown in Table 1.

**Table 1:** Clinical, biological, US and PET data

Patient	Clinical response W16	Clinical response W24	Number of PET+ joints		cSUV		Composite SUV index		Number of US+ joints		Cumulative synovial thickness (mm)		CRP (mg/l)		DAS_28_		
			J0	W16	J0	W16	J0	W16	J0	W16	J0	W16	J0	W16	J0	W16	W24
1	R	T	8	8	8.8	12.1	2.9	4	4	10	8	21.3	2.8	5.2	5,1	3.2	6.3
2	R	S	21	10	26.9	6.6	23.5	2.8	23	6	62.3	8.6	10.3	0.9	7	3.4	4.9
3	R	T	6	7	10.3	16.1	2.6	4.7	13	7	39.3	40	4.3	27.5	5.5	4.3	6.5
4	R	S	9	6	21.1	13.7	7.9	3.4	7	5	21.6	19	34.9	29.7	7	4.7	3.7
5	N-R	N-R	8	9	15.8	13.2	5.3	5	8	4	13.8	9.6	4.5	1.3	6.5	6.9	6.9
6	N-R	N-R	18	24	24.3	46.5	18.2	46.5	8	21	17.1	50.6	13.9	84.6	7.1	8.1	8.2
7	R	T	13	17	26.4	38.3	14.3	27.1	14	16	48.6	49.3	58.1	40.4	8.6	6.4	8.8
8	N-R	N-R	5	3	7.4	3.9	1.5	0.5	4	8	7.1	13.5	22	10	5.1	4.2	4.3
9	R	T	8	11	7.5	11.4	2.5	5.2	11	3	22.9	6.3	1	3.6	7.1	3.5	6.2
10	R	T	23	23	46.3	30.8	44.4	29.5	26	12	104.8	30.4	41.4	9.6	8.1	4.7	7.9
11	R	S	5	6	11.8	14.4	2.5	3.6	7	6	24	19.7	14.3	11.7	6.8	4.1	5.3
12	R	T	9	14	18.3	18.3	6.9	10.7	12	17	30.5	38.8	32.3	17.1	5.9	3.2	4.7
13	N-R	N-R	4	4	8.2	8.2	1.4	1.4	4	8	16.1	19.2	0.9	2.4	6.4	6.2	6.4
14	N-R	N-R	0	2	0	3.7	0	0.3	7	5	13.5	9.9	1.3	2.1	6.3	5.3	5.2
15	R	S	0	0	0	0	0	0	4	3	12	4.5	3	1.8	4.8	3.5	3.5

R responder, N-R non-responder, T transient, S sustained, J0 baseline, W16 week 16

### Interobserver variability

The results are shown in Table 2. Overall, there was a very good agreement between the 2 readers, both at baseline and at week 16, and both for the visual analysis and the SUVs.

**Table 2:** PET/CT analysis: interobserver agreement (*N* = 9 studies)

Joints	Baseline		W16	
	Visual analysis	SUVs	Visual analysis	SUVs
	Kappa (CI 95%)	CCC (CI 95%)	Kappa (CI 95%)	CCC (CI 95%)
Knees	0.75 (0.42, 1.00)	0.95 (0.86, 0.98)	0.85 (0.57, 1.00)	0.92 (0.80, 0.97)
Wrists	0.73 (0.38, 1.00)	0.80 (0.71, 0.86)	0.75 (0.44, 1.00)	0.93 (0.90, 0.96)
MCPs	0.77 (0.63, 0.90)	0.97 (0.96, 0.98)	0.69 (0.54, 0.84)	0.86 (0.80, 0.91)
PIPs	0.80 (0.67, 0.92)	0.99 (0.96, 1.00)	0.64 (0.49, 0.81)	0.97 (0.91, 0.99)
Global	0.79 (0.70, 0.87)	0.93 (0.61, 0.94)	0.70 (0.61, 0.80)	0.93 (0.91, 0.95)

### PET/CT data versus clinical, biological and US data

The correlation between the metabolic imaging results and the clinical results taken separately (tenderness, swelling) were poor, both at baseline and at W16 (Table 3). At baseline however, there was a significant correlation between the PET results, either visual, the cumulative SUVs or the composite SUV index on the one hand, and the comprehensive clinical assessment (DAS_28_), the CRP levels and the US results (number of joints US-positive, and cumulative synovial thickness), on the other hand (Table 4). Results are more complex at week 16, as the DAS_28_ is no longer correlated with any of the metabolic measurements, whereas the US and the metabolic assessments remain significantly correlated. The CRP remains correlated with the cSUV and the CI, but not with the number of PET-positive joints.

**Table 3:** Agreement between PET/CT (number of positive joints) and clinical parameters

Joints	Baseline: Kappa (CI 95%)		W16 Kappa (CI 95%)	
	Tenderness	Swelling	Tenderness	Swelling
Knees	0.14 (−0.19, 0.47)	0.29 (−0.04, 0.61)	0.16 (0.00, 0.33)	0.04 (−0.25, 0.32)
Wrists	0.06 (−0.25, 0.37)	0.05 (−0.31, 0.41)	−0.16 (−0.47, 0.15)	−0.24 (−0.54, 0.06)
MCPs	0.04 (−0.10, 0.19)	0.54 (0.40, 0.68)	0.16 (0.01, 0.32)	0.38 (0.22, 0.54)
PIPs	0.29 (0.14, 0.45)	0.43 (0.29, 0.57)	0.10 (0.00, 0.21)	0.36 (0.21, 0.51)
Global	0.19 (0.09, 0.28)	0.43 (0.34, 0.53)	0.12 (0.04, 0.21)	0.29 (0.19, 0.38)

**Table 4:** Correlation between the PET/CT results (number of PET-positive joints, cumulative SUV and composite SUV index, CI) and the biological, clinical and US parameters, at baseline and at week 16

	Baseline			Week 16		
	# PET+	cSUV	CI	# PET+	cSUV	CI
CRP	0.61*	0.7*	0.64*	0.39	0.76*	0.54*
DAS_28_	0.70*	0.71*	0.67*	0.14	0.33	0.24
US (# positive joints)	0.75*	0.71*	0.74*	0.55*	0.67*	0.57*
Cumulative synovial thickness (mm)	0.67*	0.76*	0.67*	0.57*	0.85*	0.66*

*indicates *P* value <0.05

### Evaluation of the response to the treatment

We used the clinical response according to the EULAR criteria as the gold standard (van Gestel et al., 1999) against which the imaging and biological parameters were confronted (Table 5). The clinical response was defined at week 16 and 24. According to these criteria, a good response was defined as a significant decrease in DAS_28_ (> 1.2) and a low level of disease activity (≤ 3.2). Non-response was defined as a decrease ≤0.6, or a decrease >0.6 and ≤1.2 with an attained DAS_28_ > 5.1. Any other scores were regarded as moderate responses. The patients were further classified in 3 groups, according to the EULAR response at week 16 and week 24: no response, transient response (response at week 16, not sustained at week 24) and sustained response (response at both week 16 and week 24). One patient (number 15) was excluded from this analysis, as he had no metabolically active disease at baseline and at week 16 (Table 1). At week 16, moderate responses were observed in 9 patients and no response in 5 patients. The 5 non-responders at week 16 remained non-responders at week 24, and only 3 had sustained clinical responses (Table 1).

**Table 5:** Concordance of metabolic, ultrasonographic and biological responses at week 16 versus clinical response at week 24, compared to baseline

	Visual PET	cSUV	CI	# US+	CST	CRP
Sensitivity	66.7	66.7	66.7	100.0	100.0	100.0
Specificity	90.9	72.7	72.7	54.6	54.6	54.6
PPV	66.7	40.0	40.0	37.5	37.5	37.5
NPV	90.9	88.9	88.9	100.0	100.0	100.0
Accuracy	85.7	71.4	71.4	64.3	64.3	64.3

Only sustained clinical responses were considered as a clinical responses; transient and absence of response were considered as a non-response. Visual PET: number of PET-positive joints; *cSUV* Cumulative SUV, *CI* Composite SUV index, *# US+* Number of US-positive joints, *CST* Cumulative synovial thickness, *CRP* C-reactive protein. Results are given in %

**Fig. 1:**
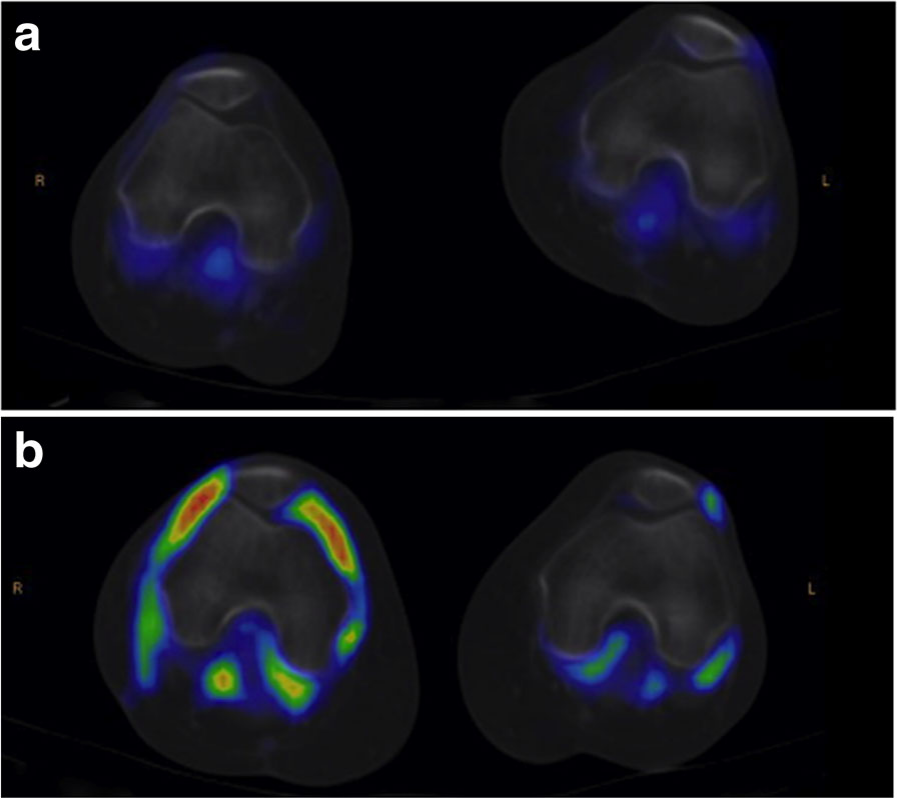
Progression of disease in a 66 year-old man. The top row (**a**, fused transaxial PET/CT images) show no abnormal uptake in the knees, whereas the PET/CT performed at week 16 show bilateral synovitis, especially in the right knee (**b**)

**Fig. 2:**
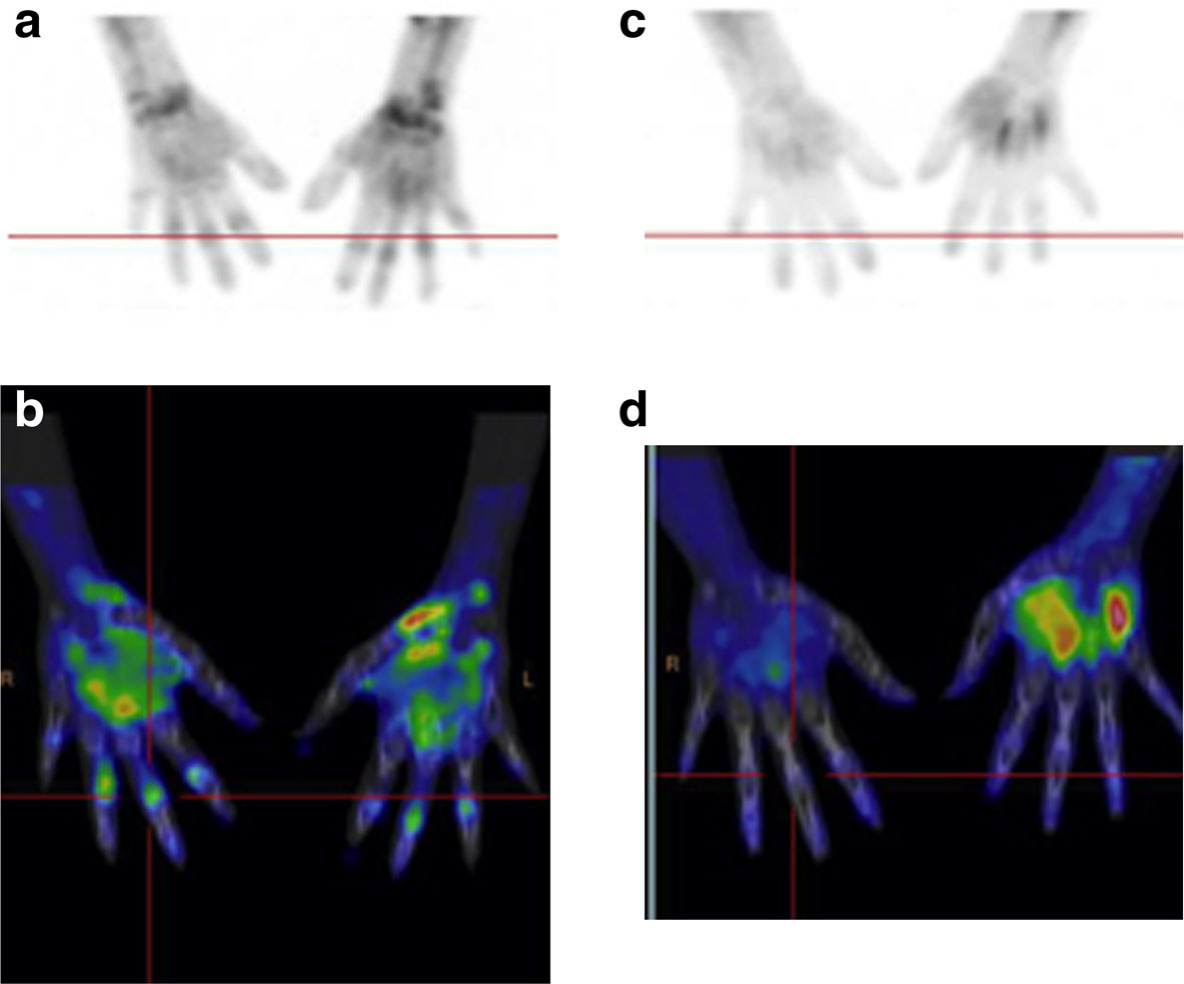
A 29 years old patient with RA diagnosed 2 years ago. At baseline, synovitis is visible in the right and left wrists and right MCP (1, 2, 3, 4), left MCP (1, 2, 3, 4), all PIP except first left PIP (**a**: 3D projection-image, **b**: coronal section). At week 16, there is a major metabolic response (**c** and **d**)

**Fig. 3:**
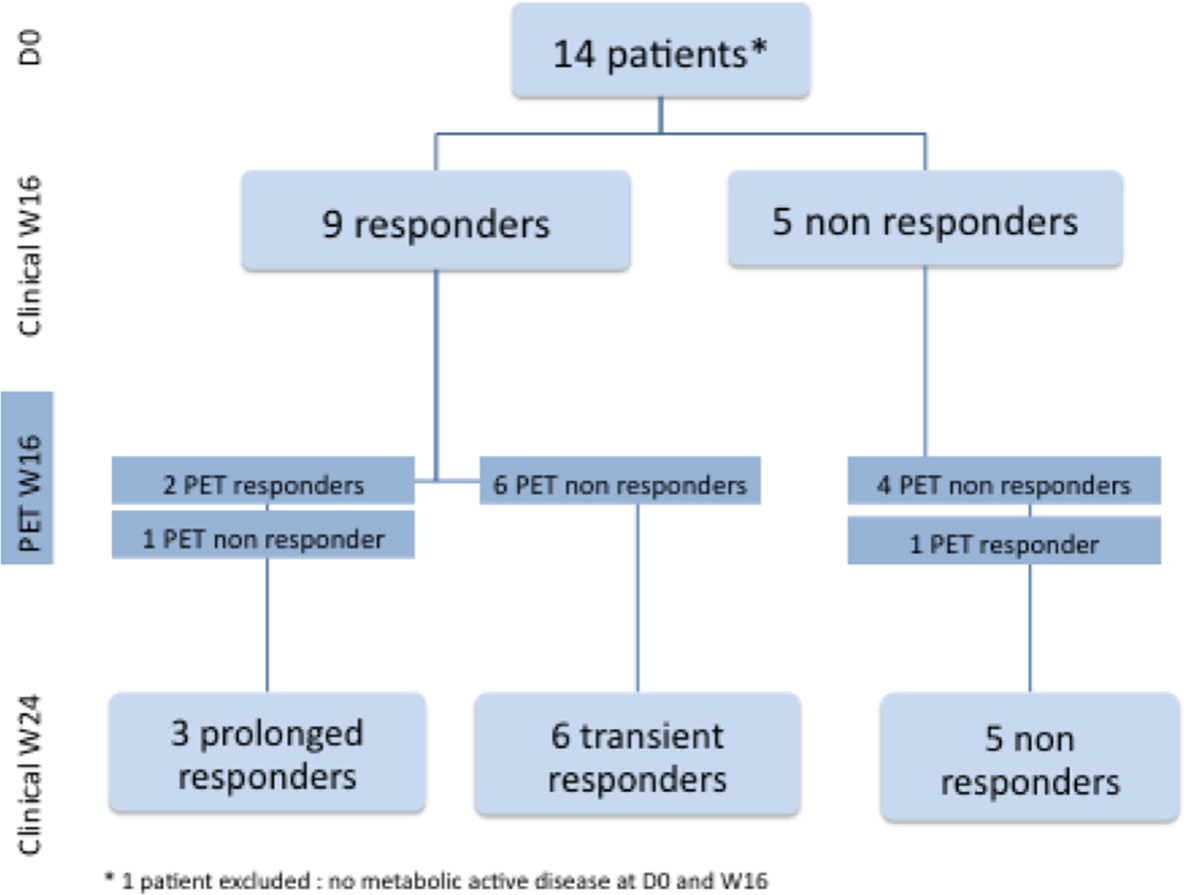
Schematic representation of metabolic and clinical responses to the treatment

A metabolic response was defined as a decrease in the number of positive joints at visual analysis, in cSUV or in the composite SUV index between baseline and week 16. Similarly, an US or biologic response was defined as decrease in the corresponding values at week 16. As shown in Table 5, the metabolic response was the most accurate for predicting the clinical response at week 24 (number of diseased joints on PET, 85.7%; cSUV 71.4%, and CI 71.4%), superior to US parameters (number of diseased joints: 64.3%; cumulative synovial thickness: 64.3%), and to serum CRP values (64.3%). Changes in the metabolic assessment (ΔCI, week 16 Vs baseline) were significantly correlated to changes in the US assessment (Δnumber of joint US+, *r* = 0.53; *P* < 0.05; and Δcumulative synovial thickness r = 0.53; *P* < 0.05). ΔSUV was also significantly correlated to changes observed in the CRP serum levels (*r* = 0.60; *P* < 0.05). Metabolic response and progression are shown in Figs. 1 and 2, respectively. The clinical and metabolic results are summarized in Fig. 3. None of the clinical, biological, US or metabolic parameters measured at baseline was predictive of the response at week 24 (data not shown).

## Discussion

The relationship between the metabolic activity assessed with ^18^F–FDG PET (Beckers et al., 2004; Beckers et al., 2006; Polisson et al., 1995; Palmer et al., 1995) or PET/CT (Kubota et al., 2011; Kubota et al., 2009; Okamura et al., 2012) and the inflammatory activity in RA has been repeatedly documented. We have previously shown that changes in SUV were significantly correlated with changes in CRP and MMP-3 serum levels, as well as with various MRI parameters, in patients who were receiving a 4-week anti-TNF-α therapy (Beckers et al., 2006). Okamura et al. showed a significant correlation between changes in SUV and changes in DAS_28_ in patients receiving, for 6 months, infliximab and etanercept, two anti-TNF-α therapies (Okamura et al., 2012). These two studies suggest that ^18^F–FDG PET/CT could be used to evaluate the response of RA patients to biologic treatments. In addition, using ^18^F–FDG PET, Elzinga et al. also showed that changes in SUV 2 weeks after initiating a treatment with infliximab correlated with the clinical evolution at 14 and 24 weeks (Elzinga et al., 2011). We applied ^18^F–FDG PET/CT to assess, and possibly predict, the response to treatment with rituximab in a highly selected population of RA patients previously resistant to anti-TNF-α therapies. First, we were able to demonstrate a high interobserver reproducibility, as both the visual analysis and SUV measurements showed high agreement, whether at baseline or during treatment. It should be noted however that the overall agreement was superior at baseline than at week 16 in particular in the small articulations such as the MCPs and PIPs. The decreased level of joint inflammation on rituximab combined with partial volume effect may explain this observation. Even though image interpretation was greatly facilitated by the CT part of the PET/CT study, the interobserver agreement was not improved compared to a series obtained with standalone PET (Beckers et al., 2004). US is generally considered a reliable tool for synovitis assessment but the interobserver agreement may be quite variable, with kappa values ranging from 0.22 to 0.868 for the B-mode, according to a recent systematic review (Cheung et al., n.d.). Further, the reproducibility of US in the therapeutic evaluation of RA is unknown.

In the present series, the metabolic results at baseline are correlated with the biological data, the DAS_28_ and the US results, which is consistent with the literature (Beckers et al., 2004; Beckers et al., 2006; Polisson et al., 1995; Palmer et al., 1995; Kubota et al., 2011; Kubota et al., 2009; Okamura et al., 2012; Elzinga et al., 2011). The principal endpoint of the study was the clinical response at week 24, according to the clinical criteria DAS_28_. Nevertheless, we added a subcategory of patients according to the clinical response at week 16: the “transient” responders, which corresponds to a response at week 16 followed by an increase in DAS_28_ at week 24. Indeed, non-responders at week 16 were not eligible for further treatment with rituximab due to Belgian regulations. On the other hand, a transient and a sustained response to a single injection of rituximab may well each represent two different patterns of evolution for the disease. According to the clinical response at week 16, 9 out of the 14 assessable patients were responders and the remaining five were non-responders. At week 24, two months later, the five non-responders remained non-responding, and 6 out of the 9 responders had flares. It must be first be noted that in our limited series, none of the parameters measured at baseline, whether clinical, biological, ultrasonographic or metabolic, were able to anticipate the response to treatment. During follow-up, all 3 patients with a sustained clinical response showed decrease in CRP levels, number of US-positive joints and cumulative synovial thickness. The clinical relevance of these parameters is however greatly limited by the very poor specificity and positive predictive value of both tests. PET/CT was less sensitive as it failed to identify a clinical responder. This patient had only 5 PET-positive joints at baseline, which increased to 6 at week 16. Both US and CRP showed a favorable evolution. A possible explanation may be the inflammatory rebound known under rituximab which may lead to overestimating the disease activity with PET, a classical observation in lymphoma treatment (Moskowitz et al., 2011), although we would expect to observe the phenomenon in a larger proportion of patients. On the contrary, 6/11 patients that eventually did not respond at week 24 showed initial clinical improvement at week 16, but 10/11 did not experience any improvement in at least one of the metabolic parameters analyzed. The most clinically relevant finding of this study is the capacity of PET to identify early on those patients who will eventually fail to respond to the treatment. Indeed, the absence of decrease in the number of hypermetabolic joints predicted the subsequent clinical failure in 91% of the cases. This may lead to a possible integration of PET/CT in the management algorithm, that would allow to move on more rapidly to another line of treatment, in hope of preventing structural damages to the joints.

Elzinga et al. found a strong correlation between the glucose metabolic rate (Mrglu) obtained by Patlak graphical analysis and the SUVs (Elzinga et al., 2011). We compared various methods for assessing the metabolic burden and found that the visual analysis was the most accurate for predicting the outcome. While the visual analysis was strongly correlated with the clinical and biological parameters at baseline, such relationship was lost at week 16 and it turned out to present the highest accuracy for predicting the clinical status at week 24. The cumulative SUVs performed poorly and a composite index taking into account the number of diseased joints fared better, but did not match the results of the visual evaluation. It seems that extinction of inflammation in target joints as evidenced by a decrease in PET-positive joints is a better predictor than a lowering of the overall inflammation, as reflected by the cumulative SUV and the composite SUV index. Contrary to Elzinga et al., we did not cluster the metacarpophalangeal joints into one and we evaluated individual joints, including the knees. This, along with differences in the patients population and treatment schemes, may contribute explaining the differences between our results and theirs. In any event, both series are quite limited and larger trials will be needed to go beyond the feasibility study. The group at Gunma University has performed extensive clinical research in RA patients treated with biologics (Okamura et al., 2012; Okamura et al., 2014; Suto et al., 2016; Suto et al., 2016; Yonemoto et al., 2016). Using the total SUVmax, i.e. the sum of the SUV in the considered joints, they showed a correlation between the evolution of the metabolic activity and the response to tocilizumab (Okamura et al., 2014). Similarly the baseline cumulative SUVmax was related to the subsequent joint destruction as assessed by X-rays (Suto et al., 2016; Yonemoto et al., 2016). In these studies however, the metabolic activity was evaluated in 8 to 12 of larger joints only, i.e. shoulders, elbows, wrists, knees, and possibly hips and ankles. Smaller joints such as the MCPs and PIPs are clinically relevant however, and we show in the present study that the metabolic activity can be recored with a high reproducibility, both using visual and semiquantitative measurements. Dedicated PET/CT devices could further refine the quality of the metabolic assessment (Chaudhari et al., 2016).

The second major observation in our series is that correlations between PET/CT and US parameters remained at week 16, and that changes observed in both technical approaches remained significantly correlated. We therefore confirm in this new set of patients the relationship between the anatomical observation of synovitis through US and the metabolic identification of these synovitis through ^18^F–FDG-PET/CT assessment, as shown earlier (Beckers et al., 2004; Beckers et al., 2006), as well as between the changes induced by rituximab, as already shown with anti-TNF-α treatments (Beckers et al., 2006). Various other methods are being tested for objectively assessing the response to rituximab treatment. High resolution US provided evidence of significant reduction in synovial hyperplasia after rituximab treatment, identifying response or resistance to rituximab and showing a link between B-cell-directed immune modulation and clinical symptoms (Ziswiler et al., 2009). Nevertheless, the evaluation of response to rituximab in this study was realized 6 months after the first administration, which seems rather late considering that the optimum rituximab re-treatment intervals is not defined and appears highly variable when based on individual’s disease progression (Smolen et al., 2007). A recent study estimated the response to treatment by performing MRI of the metacarpophalangeal joints in 10 patients, at baseline and at week 26 (Fritz et al., 2009). Decrease in volume of synovial enhancement and early rapid enhancement was associated with clinical response at 52 weeks in a subgroup of patients. Along with US and MRI, ^18^F–FDG PET/CT may provide insights into the biological activity of the disease and help guiding the selection of patients more likely to benefit from a sustained response.

Among the limitations of the present study, we acknowledge in particular that both the number of patients and the duration of the clinical follow up need to be extended. These preliminary results support the proof of concept but larger series are obviously needed to confirm the clinical relevance of this technology in rheumatoid arthritis,

## Conclusions

These preliminary data suggest that ^18^F- FDG PET/CT is a reproducible and accurate tool for evaluating disease activity in refractory rheumatoid arthritis and its non-response to rituximab. The correlation obtained with US joint assessment gives relevance to objective diseased joints through imaging techniques.

## Abbreviations

^18^F–FDG: [18F]-Fluoro-2-deoxy-2-d-glucose
CI: composite index
CRP: C-reactive protein
CST: Cumulative synovial thickness
DAS_28_: Disease activity score
ESR: Erythrocyte sedimentation rate
EULAR: European League Against Rheumatism
MCP: Metacarpophalangeal joint
MMP-3: Matrix metalloproteinase-3
MRI: Magnetic resonance imaging
PET/CT: Positron Emission Tomography/Computed Tomography
PIP: Proximal interphalangeal joint
SUV: Standardized Uptake Value
TNFα: Tumor necrosis factor-α
US: Ultrasound
VAS: Visual analogue scale
